# Diagnostic Performance of Radiography for the Evaluation of Osteoarthritis in the Equine Distal Tarsus: Comparison with Computed Tomography

**DOI:** 10.3390/ani15172522

**Published:** 2025-08-27

**Authors:** Joëlle Isabeau Steiger, Henning Richter, Brice Donati, Stefanie Ohlerth

**Affiliations:** Clinic of Diagnostic Imaging, Vetsuisse Faculty, University of Zurich, 8057 Zurich, Switzerland; joelle.steiger@uzh.ch (J.I.S.); henning.richter@uzh.ch (H.R.); brice.donati@uzh.ch (B.D.)

**Keywords:** radiography, computed tomography, osteoarthritis, equine, tarsus

## Abstract

The objective of the present study was to compare the diagnostic performance of radiography to computed tomography (CT) for the diagnosis of distal tarsal osteoarthritis. All images were interpreted and scored by 2 evaluators until a consensus was reached; they were blinded to the history, final imaging diagnosis, and results of the corresponding radiographic or CT study. On radiographs and CT images of 54 tarsi, 6 criteria (osteophyte/enthesophyte height, small/large subchondral bone radiolucencies, subchondral bone thickness, joint space narrowing) were scored in the proximal intertarsal (PIJ), distal intertarsal (DIJ), and tarsometatarsal joint (TMTJ). Compared to CT, mean radiographic scores were significantly lower for 1. small and large subchondral bone radiolucencies in all joints (*p* < 0.001–0.03); 2. subchondral bone thickness in the PIJ and DIJ (*p* = 0.03 and 0.005); and 3. enthesophyte and joint space narrowing score in the DIJ (*p* = 0.04 and 0.002). Low-to-high positive predictive values (PPV) were calculated for the individual criteria in all joints (0–100%). For the sum of scores of all criteria in the PIJ, DIJ, or TMTJ, respectively, PPVs were high (94–98%). In conclusion, radiography is a useful screening tool provided multiple criteria are assessed. However, CT offers significant advantages for the diagnosis of distal tarsal OA.

## Simple Summary

Distal tarsal osteoarthritis is a common cause of lameness in horses. Radiography represents the standard imaging technology for its diagnosis. Standing computed tomography (CT) of the equine distal extremities has become increasingly available. The objective of the present study was to compare radiography to CT for the diagnosis of distal tarsal osteoarthritis. In 54 equine tarsal joints, six criteria of osteoarthritis were scored on radiographic and CT images. In summary, for evaluation of most individual osteoarthritis criteria, radiography was inferior to CT. For the sum of scores of all criteria for each joint, though, the positive predictive values, i.e., prediction whether a particular individual will truly have the disease based on radiography, were high (94–98%). In conclusion, radiography is a useful screening tool provided multiple criteria are assessed. However, CT offers significant advantages for the diagnosis of distal tarsal OA.

## 1. Introduction

Today, osteoarthritis (OA) is defined as a disease of not just articular cartilage, but of the osteochondral unit, which is composed of articular cartilage, calcified cartilage, and subchondral and trabecular bone. These structures work synergistically to support functional loading of the joint [1,2]. Equine distal tarsal OA is common; radiography represents the modality of choice in the clinical setting for the diagnosis of equine distal tarsal OA and for monitoring its progression [3,4,5,6,7,8,9,10,11]. Cardinal radiographic findings of distal tarsal OA include osteophytes, enthesophytes, increased opacity, and thickening of the subchondral bone with loss of trabecular pattern and decreased compactospongious demarcation, subchondral bone lysis, and narrowing or loss of the radiographic joint space, as well as articular or periarticular soft tissue swelling [7,10,12,13]. Less commonly reported radiographic findings of OA in the distal tarsal joints include focal concave regions at the interface between hyaline articular cartilage and the articular calcified cartilage of the subchondral bone plate resulting in an increased distance between the mineralization fronts, central osteophytes (convex bumps at the mineralization front), and joint space mineral opacities between the mineralization fronts representing either articular or chondral mineralization [14]. 

There are few reports investigating the reliability of radiography for the detection of distal tarsal OA. Radiography was compared to degree of lameness, postmortem high detail radiographs, light microscopy histology, and scanning electron microscopy, or MRI [14,15,16,17]. Due to higher contrast resolution and lack of superimposition, computed tomography (CT) is highly accurate for the detection of bony abnormalities [18]. A few studies have investigated the use of conventional CT in the equine tarsus [19,20,21,22,23]. However, the diagnostic performance of radiography compared to CT has not yet been reported for the diagnosis of OA in the equine distal tarsal joints. 

The objective of the present study was to investigate the diagnostic performance of radiography compared to CT for the detection of single OA criteria in each distal tarsal joint (PIJ, DIJ, or TMTJ, respectively) and for the sum of scores for all single OA criteria in the PIJ, DIJ, or TMTJ, respectively. We hypothesized, 1. that frequency and severity of OA criteria are lower with radiography than with CT; and 2. that for the single OA criteria, diagnostic performance of radiography is only low to moderate compared to CT. 

## 2. Materials and Methods

### 2.1. Study Population

This retrospective study is based on multi-detector CT scans of the equine tarsus, which were acquired between 2007 and 2024 at our hospital and retrieved from our hospital’s PACS system. Out of these studies, tarsal joints were only included in the present study if first, CT image quality was good to excellent (joint in the center of the field of view without motion artefacts); second, four radiographic tarsal views ((lateromedial (LM), dorsoplantar (DP), dorsolateral–plantaromedial oblique (DLPMO), dorsomedial–plantarolateral oblique (DMPLO)) of good to excellent quality regarding contrast and positioning were available; third, time between radiographic and CT examination was less than 14 days; and fourth, body weight of the horses was 300 to 700 kg. Age, gender, breed, body weight, tarsal joint (left, right), positioning of the horse (recumbent/standing), CT unit (40-slice, 80-slice), and the final imaging diagnosis were recorded for all joints.

### 2.2. Radiographic Imaging

The four tarsal projections were acquired using different computed or digital radiography systems: FCR Fuji IP cassette type CC (resolution: pixel size 100 μm, 5 line pairs/mm) or FDR D-EVO II, Fujifilm (resolution: pixel size 150 μm, 3.3 line pairs/mm) (Fujifilm, Tokyo, Japan), Niederscherli, Switzerland, in combination with the radiographic unit Polydoros, Siemens Healthineers, Zurich, Switzerland, or Philips Optimus 65, Philips AG Healthcare, Horgen, Switzerland; Canon CXDI 810/10 (resolution: pixel size 125 μm, 4 line pairs/mm) in combination with the radiographic unit ZooMax EQ3/Gierth HF 1000, Gierth X-Ray international GmbH, Riesa, Germany. 

### 2.3. Computed Tomographic Imaging

Horses were scanned under general anesthesia and in lateral recumbency with a 40-slice CT unit (Sensation Open, Siemens, Munich, Germany) from 2007 to 2021, or under sedation, standing facing away from the machine with the tarsus gently pulled caudally through the gantry with an 80-slice CT unit (CANON Aquilion Exceed LB, Canon Medical Systems, Castle Hill, NSW, Australia) from 2022 to 2024. Transverse contiguous slices were obtained in a helical mode in all tarsal joints. Data were reconstructed into image series with 1.5 mm or 0.75 mm slice thickness in the 40-slice and 80-slice CT units, respectively, using a medium-frequency image reconstruction algorithm (soft tissue) and a high-frequency image reconstruction algorithm (bone). Computed tomographic images were transferred to a workstation and reviewed with dedicated software (IntelliSpace PACS Radiology, Version 4.4, Philips, Macquarie Park, NSW, Australia) using a multiplanar reconstruction mode and a selected bone window (window width, 2700 Hounsfield units; window level, 680 Hounsfield units). 

### 2.4. Image Analysis

All radiographic and CT studies were retrospectively evaluated in a random order. A senior board-certified radiologist (SO) with 23 years of experience in reading equine radiographs and CT images and a doctoral student/intern in large animal diagnostic imaging (JIS) evaluated all images in consensus. Consensus meant that both readers had to agree with the existence of a change. The doctoral student was previously trained to read tarsal radiographs and CT images by the senior radiologist (SO). Both assessors were unaware of the history, final imaging diagnosis, and results of the corresponding radiographic or CT study.

Six criteria were evaluated and scored in the proximal intertarsal joint (PIJ), distal intertarsal joint (DIJ), and tarsometatarsal joint (TMTJ) on radiographic and CT images: osteophyte formation, enthesophyte formation, subchondral bone thickness, small subchondral bone radiolucencies, large subchondral bone radiolucencies, and joint space narrowing. Each radiographic view was scored individually. The maximum score of each criterion was considered for the final radiographic score of each criterion. Reconstructed CT images in the dorsal, sagittal, and transverse plane were used for scoring. 

With both modalities, the height of the largest osteophyte or enthesophyte was measured in each joint and graded as nil (no osteo-, enthesophyte, score 0), small (≤1 mm, score 1), medium (>1 to ≤2 mm, score 2), and large (>2 mm, score 3). 

**Figure 1:**
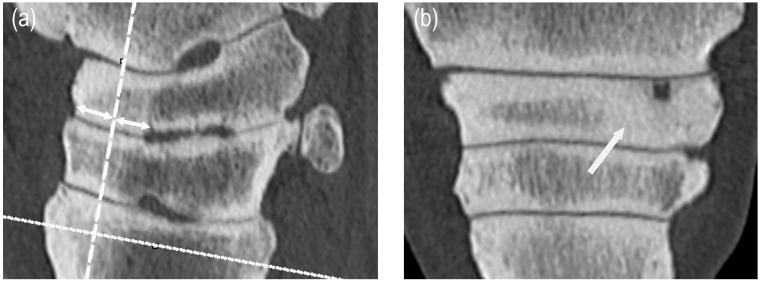
Measurements of maximum subchondral bone thickness: (**a**) the dorsal plane (dashed line) was positioned on the sagittal image perpendicular to the joint space, at 50% of the distance between the dorsal edge of the central tarsal bone (arrows) and the synovial fossae; and then (**b**) the maximum subchondral bone thickness was measured on the dorsal plane. In this example, the subchondral bone of the central tarsal bone on the medial side showed thickening of more than 4 mm and loss of the trabecular pattern (arrow).

Radiographically, maximum subchondral bone thickness was measured in each joint in the following locations: 1. LM view: between the dorsal edge of the central or third tarsal or third metatarsal bone and the synovial fossae (in the dorsal 1/3 of the joints); 2. DP, DLPMO, DMPLO views: in the central and third tarsal and third metatarsal bone, aside the synovial fossae. On CT images, maximum subchondral bone thickness was measured on dorsal plane images at standard sites, which were defined on sagittal images. For the PTJ, DTJ, and TMTJ, the dorsal plane was taken at a 90-degree angle to the joint space and at 50% of the depth of the central tarsal bone, third tarsal bone, and third metatarsal bone, respectively, between the dorsal bone surfaces and the synovial fossa (Figure 1).

For both modalities, normal maximum thickness (score 0) was defined as follows: PIJ: ≤4 mm in the central tarsal bone; DIJ: ≤3 mm in the central tarsal and third tarsal bone; TMTJ: ≤3 mm and ≤4 mm in the third tarsal bone and the metatarsal bones, respectively. In all joints, mild (score 1), moderate (score 2), and severe (score 3) subchondral bone thickening was defined as an increase of 1, 2, or 3 mm, respectively. A score of 4 was applied if thickening was >4 mm or loss of the trabecular pattern was present. 

Small and large subchondral bone radiolucencies were defined on radiographs and CT images as radiolucent areas with a maximum diameter ≤ 2 mm and >2 mm, respectively, and graded as normal/no radiolucency (score 0), one radiolucency (score 1), 2–4 radiolucencies (score 2), and >4 radiolucencies (score 3).

With both modalities, joint space narrowing was defined as no narrowing (regular and uniform height, score 0), mild narrowing (<30% of the joint width, score 1), moderate narrowing (30–70% of the joint width, score 2), severe narrowing or mild to moderate joint space collapse (narrowing > 70% of the joint width or joint space collapse ≤ 70% of the joint width or multiple bridges, score 3), and severe joint space collapse (>70% of the joint width) or subluxation (score 4). 

### 2.5. Statistical Analysis

Data sheets evaluating the presence of radiographic and CT findings were compiled for each limb. For comparison between radiographic and CT scores, each radiographic view was scored individually. The highest score was considered for the final radiographic score of each criterion. Further, a total radiographic and total CT score was calculated by adding the scores of all criteria in each joint (PIJ, DIJ, or TMTJ) for each modality.

Descriptive statistics were calculated for ordinal and continuous variables. Due to small cell counts for several ordinal variables, nonparametric tests were applied. Statistical analyses were performed for each single criterion and the total score of each single joint (PIJ, DIJ, TMTJ). Associations between the individual radiographic and CT criteria, and the total radiographic and total CT scores of each joint, respectively, were analyzed with Spearman correlation coefficients. The Wilcoxon signed-rank test was used to compare paired data, i.e., the mean of each radiographic score with the corresponding mean CT score. Diagnostic tests (sensitivity, specificity, positive and negative predictive value) were calculated for each single radiographic criterion and the total radiographic scores for each joint, with CT serving as the reference standard. For all radiographic criteria but subchondral bone thickness, a score of 0 was considered negative, and a score > 0 was classified as positive. For subchondral bone thickness, frequency of score 0 was very low in all joints; therefore, both score 0 and score 1 were treated as negative. The mean values between two independent groups, i.e., mean CT scores for the 40-slice and 80-slice CT units, were compared with the Mann–Whitney U test. Mean values between the different radiographic units could not be compared because cell counts were too small. All analyses were performed with dedicated software (IBM SPSS Statistics, Version 29.0.2.0), and *p*-values ≤ 0.05 were considered significant. Correlation coefficients of <0.5, 0.5–0.8, and > 0.8 were considered weak, moderate, and strong, respectively. Diagnostic test results of <70%, 70–90%, and >90% were considered low, moderate, and high, respectively. 

## 3. Results

Fifty-four tarsal joints of 52 horses fulfilled the inclusion criteria (24 mares, 23 geldings, 5 stallions). Age ranged from 1 to 26 years (mean and median, 10 years). A total of 26 left and 28 right tarsi were represented. Body weight ranged from 308 to 700 kg (mean, 511 kg; median, 525 kg). Thirty-two Warmblood horses, six Western horses, five Iberian horses, five ponies/cob-type horses, two Icelandic horses, and two Freiberger horses were included. In all tarsal joints, the time interval between radiographic imaging and CT was 0 to 12 days (mean, 2 days; median, 1 day). 

Thirty joints were scanned recumbent with the 40-slice CT unit, while 24 joints were examined with the 80-slice CT, of which 14 and 10 joints were scanned standing or recumbent, respectively. 

Final CT diagnosis included 2 normal joints, 9 joints with primary osteoarthritis, 23 tarsi with fractures (central tarsal bone, 14 joints; talus, splint/cannon bone, lateral malleolus, 2 joints each; calcaneus, central tarsal bone and talus, central tarsal and tarsal bone, 1 joint each), 6 tarsi with proximal suspensory desmitis, 3 tarsi each with cranial intermediate ridge osteochondrosis of the distal tibia, sequestrum or septic osteitis, one joint with a cyst-like lesion in the distal tibial metaphysis, and one tarsus each with exostosis of the second metatarsal bone or subluxation of the TMTJ, respectively. 

**Figure 2:**
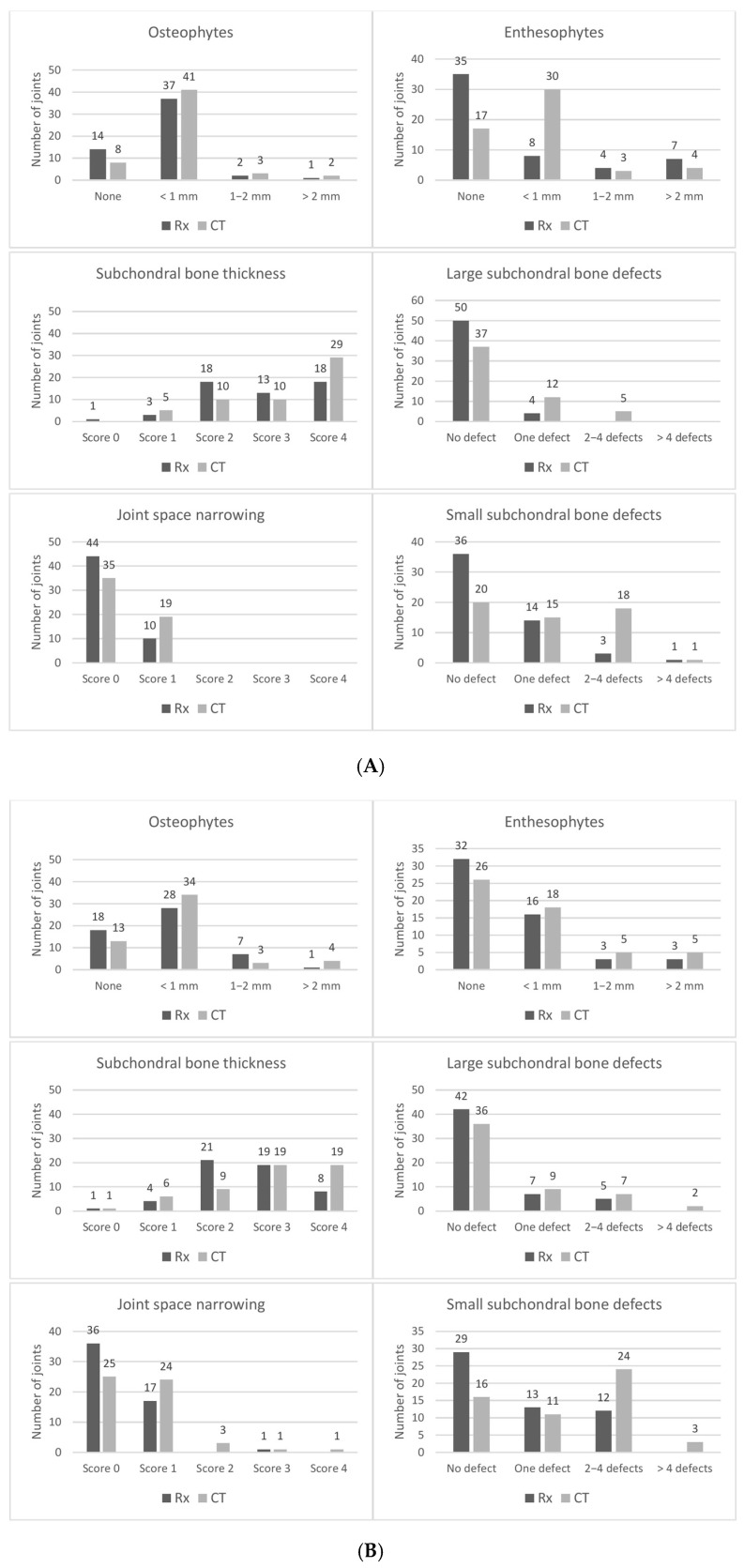

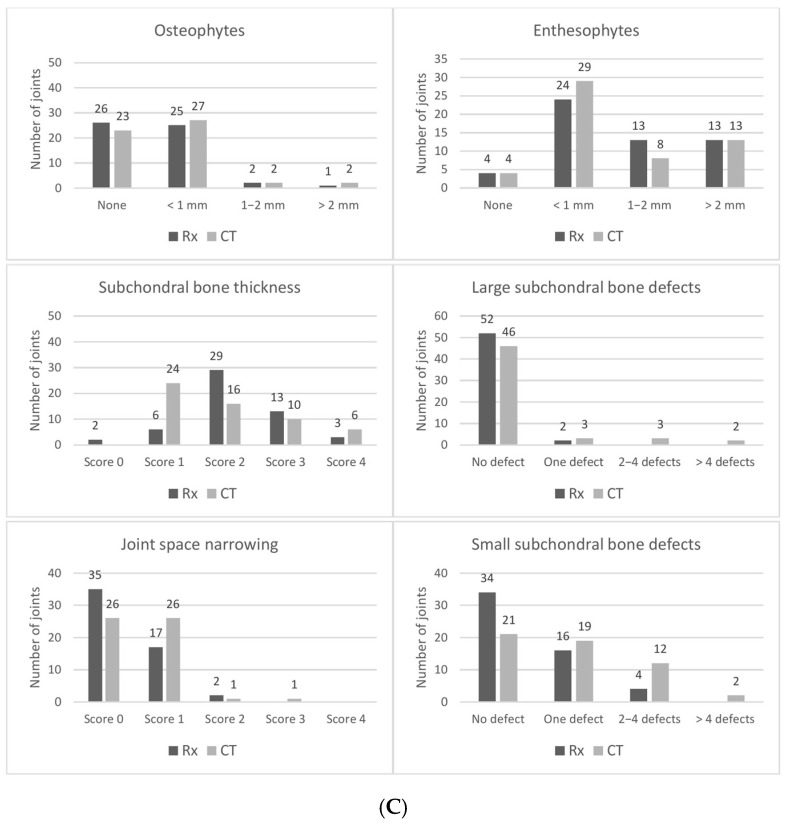
(**A**): Bar plots of the absolute frequencies of the scores for the individual radiographic and computed tomographic criteria in 54 proximal intertarsal joints. (**B**) Bar plots of the absolute frequencies of the scores for the individual radiographic and computed tomographic criteria in 54 distal intertarsal joints. (**C**) Bar plots of the absolute frequencies of the scores for the individual radiographic and computed tomographic criteria in 54 tarsometatarsal joints.

Overall, with both modalities and for all criteria but subchondral bone thickness, mild changes were more common than moderate-to-severe changes (Figure 2). A thickened subchondral bone plate was most common in the PIJ, enthesophyte formation was most prevalent in the TMTJ, and narrowing of the joint space was most common in the DIJ and TMTJ, noted with both modalities. With radiography, the proportion of normal joints was higher and the frequency of abnormal findings was lower for most criteria in all joints, compared to CT (Figure 2). 

In all joints, mean CT scores of small and large subchondral bone radiolucencies were significantly higher than mean radiographic scores (*p* < 0.001–0.03). Mean CT score of subchondral bone thickness was significantly higher in the PIJ and DIJ (*p* = 0.03 and 0.005). Mean enthesophyte and joint space narrowing score was significantly higher with CT in the DIJ (*p* = 0.04 and 0.002). Mean total CT scores of the PIJ and DIJ were significantly higher than the corresponding radiographic scores (*p* < 0.001) but not in the TMTJ (*p* = 0.1) (Table 1).

Corresponding results were found with Spearman correlation analyses (Table 1). For all joints, correlation between radiographic and CT scores was weak for small and large subchondral bone radiolucencies, osteophyte formation, and joint space narrowing (*r* = −0.152–0.386; *p* = 0.007–0.35). A weak positive correlation was also calculated for enthesophyte formation in the PIJ (*r* = 0.489; *p* < 0.001) and subchondral bone thickness in the TMTJ (*r* = 0.21; *p* = 0.13). Moderate positive correlation values were obtained for subchondral bone thickness (*r* = 0.544 and 0.523; *p* < 0.001) in the PIJ and the DIJ, and for enthesophyte formation (*r* = 0.523 and 0.601; *p* < 0.001) in the DIJ and TMTJ. Correlations between the total radiographic and CT scores of the PIJ, DIJ, or TMTJ were moderate (*r* = 0.534–0.65; *p* < 0.001).

**Figure 3:**
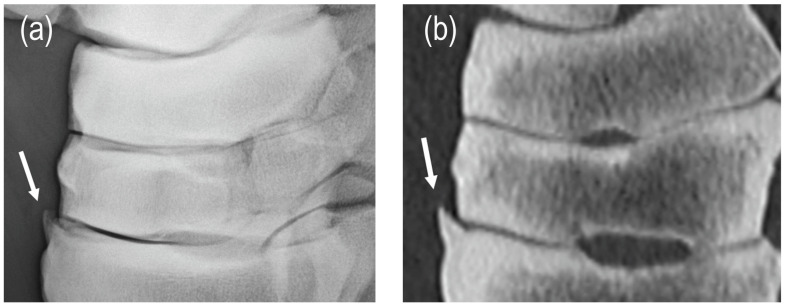
Note the clearly identifiable enthesophyte (arrows) at the dorsal margin of the tarsometatarsal joint on the radiographic (**a**) and CT image (**b**).

**Figure 4:**
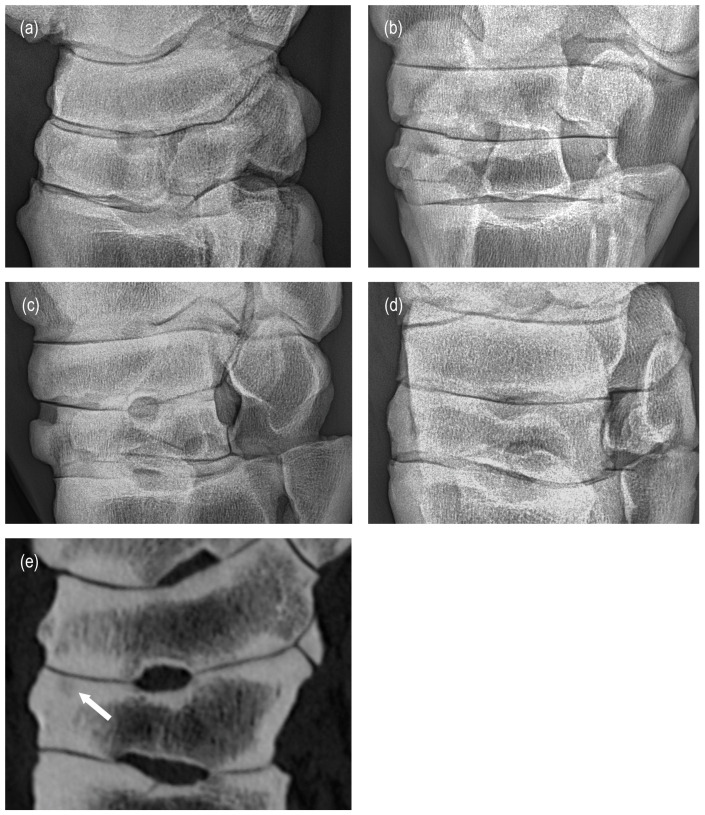
In this tarsal joint with a final diagnosis of osteochondrosis in the distal tibia, a large subchondral bone radiolucency in the third tarsal bone (arrow) was not identified radiographically ((**a**) lateromedial, (**b**) dorsoplantar, (**c**) dorsolateral–plantaromedial and (**d**) dorsomedial–plantarolateral view) but clearly seen on CT ((**e**) sagittal plane image). Subchondral bone thickness > 4 mm and loss of the trabecular pattern were diagnosed with both modalities.

**Figure 5:**
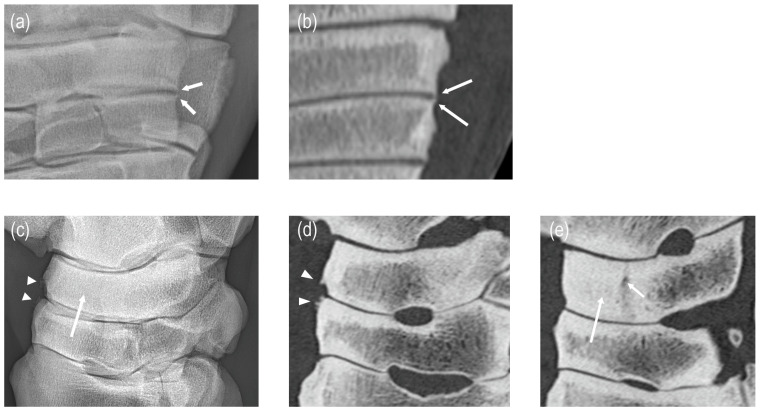
Two cases in which osteoarthritic changes were clearly visible on radiographic and CT images: (**a**) small osteophytes (arrows) at the lateral aspect of the distal intertarsal joint (DIJ) are similarly depicted on a dorsoplantar radiographic view and on a corresponding dorsal plane CT image (**b**); enthesophytes (arrowheads) are visible on the dorsal aspect of the DIJ on the lateromedial radiographic view (**c**) and a sagittal CT image (**d**); equivalent subchondral bone thickness of the central tarsal bone (long arrow) is apparent on a radiographic (**c**) and sagittal CT image (**e**). Notably, the large subchondral bone radiolucency of the central tarsal bone (short arrow, (**e**)) is not discernible radiographically (**c**).

Diagnostic test values were calculated for each single radiographic criterion (Table 2). A high proportion of false-negative joints combined with a rather low number of true-positive joints was noted for most single criteria in all joints, resulting in low-to-moderate diagnostic test values. Enthesophyte formation in the TMTJ was the only criterion with high sensitivity (94%) and a high positive predictive value (94%) (Figure 3). A high positive predictive value was also calculated for subchondral bone thickness in the DIJ (93%), but sensitivity was low (66%). Moderate sensitivity values were calculated for osteophytes in the PIJ and DIJ (78% and 71%), whereas all other single criteria had low-to-borderline-moderate sensitivity values (0–68%). In all joints, sensitivity was the lowest for large subchondral radiolucencies (0–39%) with corresponding highest specificity and negative predictive values (86–96% and 70–85%) (Figure 4). In the PIJ, sensitivity was also very low for joint space narrowing (11%). For all other criteria, specificity values were low-to-moderate (25–88%). Moderate positive predictive values were achieved for osteo- and enthesophytes and small subchondral radiolucencies in the PIJ and the DIJ (77–90%) (Figure 5), for subchondral bone thickness in the PIJ (84%), joint space narrowing in the DIJ (72%), and small subchondral radiolucencies in the TMTJ (75%), respectively. Negative predictive values were low (25–66%) for all criteria in all joints except for large subchondral radiolucencies in all joints (70–85%) and subchondral bone thickness in the TMTJ (81%). For the total radiographic score for the PIJ, DIJ, or TMTJ, the number of true-positive joints was very high, resulting in high sensitivity and positive predictive values in all joints (94–98% and 98–100%). However, due to the low number of true-negative joints, specificity and negative predictive values were low in all joints (50% and 0–50%) or could not be calculated at all.

Mean CT scores were also compared between the CT technologies used in the present study. Mean subchondral bone thickness and enthesophyte score of the DIJ were significantly higher with the 80-slice unit (*p* = 0.005 and 0.029), whereas joint space narrowing scores of the DIJ and TMTJ were significantly higher with the 40-slice unit (*p* < 0.001).

## 4. Discussion

Radiography represents the standard imaging technology for the examination of equine tarsal OA because of its wide availability, feasibility, and low costs. The performance of radiography for the diagnosis of equine distal tarsal OA has been sparsely reported [14,16,17] and has not been compared to CT. Computed tomography also represents an X-ray imaging technology. Therefore, comparison between radiography and CT seems reasonable. As hypothesized by the authors, frequency and severity of abnormal findings were, in general, higher with CT than with radiography. Weak to at best moderate positive correlations were calculated for the single criteria as well as for the total score of each joint. The second hypothesis of this study was also confirmed: for most single OA criteria, the diagnostic performance of radiography was only low-to-moderate compared with CT. These results correspond to previously published studies. One study found little correlation between duration or degree of lameness and severity of the radiographic findings in horses with distal tarsal OA [15]. In another study, statistical agreement between clinical radiographs and postmortem high-detail radiographs was poor to moderate for subchondral bone plate irregularities and joint margin changes [16]. In the distal intertarsal joint of Icelandic horses, sensitivity values of 46–88% and specificity values of 65–100% were reported for the radiographic detection of joint margin osteophytes, mineralization front defects, or pooled results, compared to light microscopy histology or scanning electron microscopy [14]. Another study compared radiography and magnetic resonance imaging (MRI) in disorders of the distal tarsal bones. A small group of horses with OA was also included. The authors concluded that radiography was not very reliable for the detection of osteoarthritis (5 of 8 horses) and subchondral bone sclerosis (9 of 16 horses) [17]. Lower performance of radiography compared to CT may be well explained with CT representing a cross-sectional imaging technology, free of superimposition of structures [18]. In complex anatomic regions, such as the composite tarsal joints, this represents a major advantage for the identification of normal or abnormal bone. Moreover, the rather rigid, low-motion distal tarsal joints exhibit narrow, obliquely running joint spaces. Radiographic projection of such without superimposition in all aspects of the joint space is difficult or almost impossible to achieve. Therefore, interpretation is challenging [10,24]. Subchondral bone abnormalities may be overlooked radiographically due to summation with adjacent healthy bone or sclerosis [21]. The anatomic conditions may explain the significantly lower radiographic scores of small and large subchondral bone radiolucencies in all distal tarsal joints of the present study population. Results were slightly better for measured variables, i.e., osteo- and enthesophyte height and subchondral bone thickness. Osteo- and enthesophytes in the distal tarsal joints occur primarily at the articular margins and less commonly in the central articular aspects [7,8,12,16,25]. These are probably depicted radiographically more accurately as marginal changes are more likely to be projected free of superimposition, noting that four projections are available, as in the present study. In addition, the extent of osteo- and enthesophyte formation and subchondral bone thickening was not quantified; only the maximum height or thickness in relation to the entire joint was recorded in the present study.

Enthesophyte score in the TMTJ represented the overall most common abnormality, with also the highest scores. It was the only criterion with high sensitivity and positive predictive values. For all other criteria, the higher prevalence of mild changes combined with infrequent moderate-to-severe changes may, in part, explain their lower sensitivity and positive predictive values. It may be hypothesized that the diagnostic performance of radiography may have improved with a larger study population and more balanced groups of scores. While sensitivity and specificity are generally considered stable features of a given test, positive and negative predictive values depend on the pre-test probability (i.e., probability of the presence of the disease before a diagnostic test), which is determined by baseline risk factors such as disease prevalence [26].

Many single criteria of all joints had moderate positive predictive values (72–90%). Only two criteria had high positive predictive values (95% and 94%). In other words, for these single criteria, approximately 3–4 out of 5 abnormal joints were radiographically predicted correctly. For the total scores in each joint, the number of true-positive joints was very high, resulting in high sensitivity and positive predictive values in all joints. This result shows that even if the same changes were not seen on radiographs as on CT images, at least one other change was present radiographically, reflecting the proportion of true-positive results in a joint very well. Vice versa, due to the low number of true-negative joints, specificity and negative predictive values were very low in all joints. This indicates that if no radiographic changes were present, there may well have been changes in CT.

It is interesting to note that in all joints, large subchondral bone radiolucencies had the lowest true-positive rates and highest false-negative rates. In the authors’ experience, these were difficult to identify due to their central location within the tarsal bones. In contrast, many of the small subchondral bone radiolucencies represented mineralization front defects [14]. They are located close to the radiographic joint space, i.e., the interface between hyaline articular cartilage and the articular calcified cartilage of the subchondral bone plate. Consequently, they were more easily detectable. Large subchondral bone radiolucencies in the equine fetlock have also been recently reported to be missed radiographically in a large proportion of affected horses [27,28]. In the equine distal limb, MRI and CT were superior for the diagnosis of subchondral bone disease [29]. The etiopathogenesis of large subchondral bone radiolucencies identified in the present study remains unclear. An association between osteoarthritis and the formation of subchondral bone cysts has been described in human medicine [30,31]. To date, no reports have described an association between osteoarthritis and the development of large subchondral bone radiolucencies in the equine distal tarsus. Three major theories have been proposed regarding the formation of large subchondral bone radiolucencies [32]. First, the hydrodynamic theory postulates that synovial fluid enters the subchondral bone through a full-thickness cartilage defect, driven by intra-articular pressure due to synovitis. This may result in pressure-induced necrosis and subsequent bone resorption [31,33]. Second, the contusion theory attributes subchondral radiolucencies, bone marrow edema, or necrosis to traumatic impact between opposing articular surfaces, with or without associated cartilage damage [34]. Third, focal ischemia within the epiphyseal growth cartilage without articular cartilage damage results in chondronecrosis or vascular dilation, ultimately leading to cyst formation in the context of osteochondrosis [35]. In the present study, assessment of the condition and integrity of the articular cartilage was not possible, as MRI was not available, and CT imaging does not permit visualization of cartilage in the absence of intra-articular contrast. In the present study, chronic fractures of the central tarsal bone were frequent and often accompanied by large subchondral bone radiolucencies. On the other hand, large subchondral bone radiolucencies were also observed in subjects without a fracture. The etiopathogenesis of these lesions may likely be similar to sagittal groove injuries in the first phalanx, which are characterized by stress-related, short, incomplete fractures, and/or large subchondral bone radiolucencies [27,32,36].

A certain number of false-positive joints were found for each criterion in each joint. There are probably various reasons for this. Oblique projection and superimposition of articular margins, joint spaces, bony contours, and spongious/compact bone may have mimicked osteophytes, enthesophytes, joint space narrowing, subchondral bone thickening, or subchondral radiolucencies. For example, both osteo- and enthesophytes commonly occur in the dorsal aspect of the TMTJ. Their frequent close proximity to each other caused difficulties in differentiating them radiographically. Additionally, if a score greater than zero was given in a single radiographic projection, with the remaining projections being normal, this was included in the overall result. This contrasts with conventional radiographic imaging practice, which considers a finding to be more real and relevant if it is visible on several projections [37].

This study had several limitations. First, the study population included very few normal, healthy joints, and fractures, particularly of the central tarsal bone, were overrepresented. The high number of central tarsal bone fractures may also explain that a thickened subchondral bone plate and narrowing of the joint space were very common in the DIJ of the subjects of the present study. Horses were preselected for CT in the present study. At our institution, according to common practice, radiography is performed as a first-line imaging modality in horses with tarsal pain. The decision whether a horse undergoes a tarsal CT scan is usually made when radiographs are inconclusive, a fracture is suspected, or CT is needed for surgical planning. Second, although the image quality was subjectively addressed and subjectively considered equivalent between the different systems by an experienced radiologist, the radiographic and CT examinations were not standardized. Different systems were used with varying resolution, slice thickness, or algorithms, respectively. Further, the 80-slice CT unit included both standing and recumbent positions. The radiographic systems, as well as CT positioning, could not be compared due to a small sample size. Both CT units could be statistically compared, revealing some significant differences. This is likely due to the population itself (different prevalence), but a device-related cause cannot be ruled out. The latter needs to be investigated in a follow-up study using both CT systems on the same joints. Thirdly, grading of subchondral bone thickness was based on the recent literature, and our values were chosen very strictly in the lower reported ranges [38]. Under these circumstances, normal subchondral bone thickness was rarely observed in the present study. Depending on the exercise intensity and type, score 1 as defined in this study may still be considered normal according to the reference literature. Therefore, score 0 and score 1 were considered negative for the calculation of the test values. Fourthly, with our evaluation scheme, even very small changes were evaluated, and their clinical significance remains unclear, especially given the retrospective nature of the present study. 

## 5. Conclusions

In the present study, frequency and severity of single OA criteria in the equine distal tarsal joints were, in general, lower with radiography than with CT. Diagnostic performance of radiography was inferior to CT for all single OA criteria. Especially for the detection of large subchondral bone radiolucencies, performance of radiography was low. Radiography is a useful screening tool provided multiple criteria are assessed. However, CT offers significant advantages for the diagnosis of distal tarsal OA.

## Figures and Tables

**Table 1:** Results of 1. the descriptive statistics, 2. the correlation analyses between the radiographic and corresponding computed tomographic scores, and 3. the Wilcoxon signed-ranked tests comparing the mean value of a radiographic score with the corresponding CT score, in 54 tarsal joints (MN: mean; MD: median; SE: standard error; SD: standard deviation; RX: radiography; CT: computed tomography; * *p*-value < 0.05).

		MN	MD	SE	SD	Wilcoxon Signed-Ranked Test	Spearman Correlation Coefficient
Proximal intertarsal joint							
Osteophytes	RX	0.81	1.00	0.080	0.585	*p* = 0.06	0.305 (*p* = 0.03) *
	CT	0.98	1.00	0.081	0.598		
Enthesophytes	RX	0.69	0.00	1.047	1.079	*p* = 0.14	0.489 (*p* < 0.001) *
	CT	0.89	1.00	0.111	0.816		
Subchondral bone thickness	RX	2.83	3.00	0.142	1.033	*p* = 0.03 *	0.544 (*p* < 0.001) *
	CT	3.17	4.00	0.142	1.042		
Small subchondral bone radiolucencies	RX	0.43	0.00	0.094	0.690	*p* < 0.001 *	0.278 (*p* = 0.04) *
	CT	1.00	1.00	0.121	0.890		
Large subchondral bone radiolucencies	RX	0.07	0.00	0.036	0.264	*p* = 0.001 *	0.131 (*p* = 0.35)
	CT	0.41	0.00	0.090	0.659		
Joint space narrowing	RX	0.19	0.00	0.053	0.392	*p* = 0.07	−0.152 (*p* = 0.27)
	CT	0.35	0.00	0.066	0.482		
Distal intertarsal joint							
Osteophytes	RX	0.83	1.00	0.098	0.720	*p* = 0.22	0.350 (*p* = 0.009) *
	CT	0.96	1.00	0.106	0.776		
Enthesophytes	RX	0.57	0.00	0.114	0.838	*p* = 0.04 *	0.523 (*p* < 0.001) *
	CT	0.80	1.00	0.131	0.959		
Subchondral bone thickness	RX	2.55	3.00	0.125	0.911	*p* = 0.005 *	0.523 (*p* < 0.001) *
	CT	2.91	3.00	0.145	1.069		
Small subchondral bone radiolucencies	RX	0.69	0.00	0.112	0.820	*p* = 0.001 *	0.364 (*p* = 0.007) *
	CT	1.26	1.50	0.130	0.955		
Large subchondral bone radiolucencies	RX	0.31	0.00	0.087	0.639	*p* = 0.03 *	0.386 (*p* = 0.004) *
	CT	0.54	0.00	0.117	0.862		
Joint space narrowing	RX	0.37	0.00	0.081	0.592	*p* = 0.002 *	0.343 (*p* = 0.01) *
	CT	0.69	1.00	0.112	0.820		
Tarsometatarsal joint							
Osteophytes	RX	0.59	1.00	0.090	0.659	*p* = 0.39	0.312 (*p* = 0.02) *
	CT	0.69	1.00	0.098	0.722		
Enthesophytes	RX	1.65	1.00	0.127	0.935	*p* = 0.41	0.601 (*p* < 0.001) *
	CT	1.56	1.00	0.129	0.945		
Subchondral bone thickness	RX	2.17	2.00	0.117	0.849	*p* = 0.11	0.210 (*p* = 0.13)
	CT	1.89	2.00	0.131	0.965		
Small subchondral bone radiolucencies	RX	0.44	0.00	0.086	0.634	*p* = 0.001 *	0.244 (*p* = 0.08)
	CT	0.91	1.00	0.119	0.875		
Large subchondral bone radiolucencies	RX	0.04	0.00	0.026	0.191	*p* = 0.03 *	−0.081 (*p* = 0.56)
	CT	0.28	0.00	0.100	0.738		
Joint space narrowing	RX	0.39	0.00	0.077	0.564	*p* = 0.09	0.228 (*p* = 0.10)
	CT	0.57	1.00	0.086	0.633		
Total scores							
Total score proximal intertarsal joint	RX	4.96	5.00	0.290	2.128	*p* < 0.001 *	0.650 (*p* < 0.001) *
	CT	6.80	7.00	0.300	2.201		
Total score distal intertarsal joint	RX	5.28	5.00	0.339	2.491	*p* < 0.001 *	0.631 (*p* < 0.001) *
	CT	7.15	6.50	0.458	3.367		
Total score tarsometatarsal joint	RX	5.24	5.00	0.272	1.999	*p* = 0.1	0.534 (*p* < 0.001) *
	CT	5.89	5.00	0.413	3.039		

**Table 2:** Results of the diagnostic tests for the single radiographic criteria and the total radiographic scores for the proximal intertarsal, distal intertarsal and tarsometatarsal joint, compared to computed tomography (TP: number of true-positive joints; FP: number of false-positive joints; FN: number of false-negative joints; TN: number of true-negative joints; PPV: positive predictive value; NPV: negative predictive value; n.a.: not assessable).

	TP	FP	FN	TN	Sensitivity (%)	Specificity (%)	PPV (%)	NPV (%)
Proximal intertarsal joint								
Osteophytes	36	4	10	4	78	50	90	29
Enthesophytes	17	2	20	15	46	88	89	43
Subchondral bone thickness	26	5	12	10	68	67	84	46
Small subchondral radiolucencies	14	4	20	16	41	80	78	44
Large subchondral radiolucencies	2	2	15	35	12	95	50	70
Joint space narrowing	2	8	17	27	11	77	20	61
Distal intertarsal joint								
Osteophytes	29	7	12	6	71	40	81	33
Enthesophytes	17	5	11	21	61	81	77	66
Subchondral bone thickness	25	2	13	13	66	87	93	50
Small subchondral radiolucencies	21	4	17	12	55	75	84	42
Large subchondral radiolucencies	7	5	11	31	39	86	58	74
Joint space narrowing	13	5	16	20	45	80	72	56
Tarsometatarsal joint								
Osteophytes	19	9	12	14	61	61	68	54
Enthesophytes	47	3	3	1	94	25	94	25
Subchondral bone thickness	6	10	7	30	46	75	38	81
Small subchondral radiolucencies	20	5	23	17	45	76	75	47
Large subchondral radiolucencies	0	2	8	44	0	96	0	85
Joint space narrowing	13	6	15	20	46	77	68	57
Total scores								
Total score proximal intertarsal joint	51	0	3	0	94	n.a.	100	0
Total score distal intertarsal joint	51	1	1	1	98	50	98	50
Total score tarsometatarsal joint	53	0	1	0	98	n.a.	100	0

## Data Availability

Data are contained within the article.
